# Suicide-related internet use of mental health patients: what clinicians know

**DOI:** 10.1192/bjo.2024.793

**Published:** 2024-11-05

**Authors:** Lana Bojanić, Jessica Kenworthy, Tamara Moon, Pauline Turnbull, Saied Ibrahim, Navneet Kapur, Louis Appleby, Isabelle M. Hunt, Sandra Flynn

**Affiliations:** National Confidential Inquiry into Suicide and Safety in Mental Health (NCISH), University of Manchester, Manchester, UK; Department of Psychology, University of Staffordshire, Staffordshire, UK; Camden and Islington NHS Foundation Trust, London, UK; Centre for Mental Health and Safety, Manchester Academic Health Sciences Centre, University of Manchester, Manchester, UK; Mersey Care NHS Foundation Trust, NIHR Greater Manchester Patient Safety Research Collaboration, University of Manchester, Manchester, UK

**Keywords:** Suicide, disclosure, internet, suicide methods, clinican training.

## Abstract

**Background:**

Suicide-related internet use (SRIU), defined as internet use related to one's own feelings of suicide, can be both a risk and protective factor, especially for isolated individuals. Despite its influence on suicidality, clinicians face challenges in assessing SRIU because of the private nature of internet usage. Current recommendations on enquiring about SRIU in a clinical setting concern mostly young people.

**Aims:**

To address the gap in understanding SRIU among patients of all ages, this study aims to explore mental health clinicians’ experiences, attitudes and beliefs regarding enquiring about SRIU, as well as the risks and benefits it presents in the assessment and management of patients. Finally, the study aims to establish the role SRIU potentially plays in the assessment and management of patients.

**Method:**

Twelve clinicians practising at secondary mental health services in England participated in interviews. Thematic analyses were used for data interpretation.

**Results:**

Clinicians who participated in interviews rarely initiate discussions on SRIU with their patients despite considering this an important factor in suicidality. Age of both patients and clinicians has the potential to influence enquiry into SRIU. Clinicians recognise the potential benefits of patients finding supportive online communities but also express concerns about harmful and low-quality online content related to suicide.

**Conclusions:**

Integrating SRIU enquiry into standard clinical practice, regardless of the patient's age, is an important step towards comprehensive patient care. Broader training for clinicians on enquiring about online behaviours is essential to mitigate potential risks and harness the benefits of SRIU in mental health patients.

Suicide-related internet use (SRIU) is defined as the ‘use of internet for reasons relating to an individual's own feelings of suicide’.^1^ Within this broad definition, SRIU can be both a risk and protective factor for suicide. Durkee et al^2^ noted that this duality was most evident for isolated and vulnerable individuals who are most susceptible to the negative aspects of the internet yet can also benefit the most from internet use. Anonymity among a community of internet users can provide opportunities to openly discuss difficult and often stigmatising topics, but can also result in discussing lethal methods or encouragement to see plans of suicide through.

In England, following the death by suicide of a 14-year-old girl, the well-publicised coroner's inquest concluded that her death was influenced, in part, by ‘negative effects of online content’, noting that she had accessed suicide and self-harm-related content online, which exacerbated her depression.^3^ This inquest conclusion has affirmed SRIU as a factor affecting suicidality, as can be seen in the resent Suicide Prevention Strategy for England that calls for improved online safety and moderating of suicide-related content to help reduce suicide rates.^4^ A growing body of research in the UK has shown a moderate correlation between searching for suicide-related search terms online and suicide rates.^5–7^ Furthermore, SRIU has been reported to influence the choice of suicide methods;^8^ this was found to be the case in mental health patients who died by suicide and had also engaged in SRIU.^9^ The same research has shown that, even though more prevalent in young patients, SRIU was present across all age groups in deceased mental health patients, with most of those who engaged in SRIU being between 25 and 44 years of age.^9^

With internet usage being a mostly private behaviour, it is challenging for clinicians to establish whether their patients are engaging in SRIU; yet, such knowledge may be important in risk assessment and safety planning. In studies of young people and clinicians on mental health consultations and digital technology use in general, clinicians and young people agreed that exploring digital technology use and its impact on mental health should be a standard part of the assessment.^10,11^ In addition, a qualitative study by Padmanathan et al^12^ found that clinicians supported enquiring about SRIU in patients, as they believed it may be indicative of increased risk and intent. Hawton et al^13^ advise that exacerbation in dynamic factors can indicate warning signs for suicidality and suggest exploring those factors repeatedly with the patient throughout the episode of care.

Even though there is a degree of consensus on the importance of this enquiry, current evidence suggests clinicians rarely or never assess internet activity directly.^10–12^ A number of barriers to this enquiry have been listed, including lack of guidance, time concerns about incorporating such enquiries into one-off assessments and the potential risk of ‘introducing’ patients to harmful SRIU.^10,14^ Recommendations from several studies and a recent policy report on internet use and suicide prevention assert that comprehensive training is needed to facilitate disclosure and start conversations with patients on their internet use, including SRIU.^10–12,14–17^ Recently, Biddle et al^15^ developed good practice guidelines for conversations about online harms with young people and concluded that such conversations should be initiated with all young people presenting to mental health services, especially those with a history of self-harm and suicidal thoughts. However, current knowledge concerns SRIU in children and young people only, despite this behaviour appearing to be common across all age groups.^9^

More insight is needed into clinicians’ experiences with SRIU in their patients regardless of age, their attitudes and beliefs regarding it and how disclosure currently occurs. This study aims, first, to explore mental health clinicians’ experiences and attitudes towards enquiring about SRIU with patients; second, to explore clinicians’ perspectives and attitudes towards risks and benefits of SRIU and, third, to establish the role SRIU potentially plays in the assessment and management of patients.

## Method

### Participants and setting

The main inclusion criteria for participants was currently working in National Health Service (NHS) secondary mental healthcare in England. Participants were recruited in 2022 through research and development departments at their respective trusts. Recruitment ceased when consistency within themes was reached; in total, 12 participants were recruited. Because of the open and opt-in nature of recruitment, the exact number of potential participants was unknown. Five participants were clinical psychologists, three were consultant psychiatrists, three were mental health nurses and one was a care support worker. Six participants were women and six were men, with the median age of all participants being 36 (range: 25–57, interquartile range (IQR) = 12.3). All participants had carried out suicide risk assessments as part of their role and four worked primarily with suicidal patients and those who self-harm. Whilst participants were asked explicitly about *suicide-*related internet use, some also mentioned instances of self-harm-related internet use. Mentions of self-harm instances have been included in the results for two reasons. Suicidal intent, the key differentiator between self-harm and suicide, is hard to determine and varies over time,^18^ especially in second-hand clinician reports. In addition, even those with low suicidal intent for self-harm are at risk for subsequent suicide,^19^ justifying inclusion of self-harm-related internet use instances in the study results.

### Data collection

A single semi-structured interview was carried out with each clinician to explore their experiences, thoughts and beliefs regarding SRIU of mental health patients they have previously treated or were currently treating. Written informed consent was obtained from all participants. Basic demographic data (gender, age) and the professional role of the clinicians were also collected. Previous experience with patients’ SRIU was not a prerequisite to participate, with SRIU of mental health patients defined as any use of the internet connected to the patients’ suicidality. The interview topic guide was developed by L.B. and reviewed by I.H. and S.F. (for full interview topic guide see Supplementary Appendix 1 available at https://doi.org/10.1192/bjo.2024.793). In addition, the interview topic guide was independently reviewed by a practising clinician (expert by experience). Questions in the interview topic guide covered general views on disclosure of SRIU and thoughts on its benefits and risks for mental health patients (see Table 1). Before the interview, participants were informed of the study's aims and gave written informed consent. All interviews were carried out online by the primary researcher (L.B.), audio recorded and transcribed verbatim. The authors assert that all procedures contributing to this work comply with the ethical standards of the relevant national and institutional committees on human experimentation and with the Helsinki Declaration of 1975, as revised in 2008. Ethical permission for this study was obtained from the NHS Research Ethics Committee and Health Research Authority and Health and Care Research (21/WA/0212).

**Table 1:** Themes and subthemes

1. SRIU clinicians have encountered	1.1. SRIU was common and varied 1.2. Researching suicide methods
2. Disclosure of SRIU	2.1. SRIU is not enquired about 2.2. Spontaneous disclosure 2.3. Asking about SRIU: a standard/mandatory question
3. Individual differences influence disclosure	
4. Awareness of researching means online as an opportunity to intervene	
5. Duality of SRIU	5.1. Online communities as ‘safe spaces’ for patients 5.2. Using the internet instead or as a supplement to offline service provision 5.3. Encountering harmful content when searching for help or inadvertently

SRIU, suicide-related internet use.

### Data analysis

Thematic analysis was conducted^20^ with interview questions used deductively to inform the initial codes. There were three stages to the analysis. First, the lead author (L.B.) independently coded the interview transcripts, developed the themes and interpreted them. Additional codes and context were captured after familiarisation with the transcripts and immersion in the data. Second, a sample of data (six transcripts) was independently coded by J.K. and complete intercoder consistency was established through joint review for convergences of codes by J.K. and L.B. Third, final identified themes were checked against codes and relevant transcript excerpts by L.B. to ensure accurate reflection of the participants’ experiences. The thematic structure and illustrative quotes were finalised and agreed upon by all co-authors. NVivo version 12 Plus for Windows (Lumivero, Denver, USA; see https://lumivero.com/products/nvivo/) was used for data management and coding.

## Results

There were five themes identified, with eight subthemes. An overview of the themes and subthemes is presented in Table 1. We want to clarify that the themes represent clinicians’ assumptions and beliefs about patients’ SRIU and may not fully capture the reality experienced by patients.

### Theme 1: SRIU clinicians have encountered

The first theme illustrated perceived prevalence and types of SRIU clinicians encountered in their patients.

#### SRIU was common and varied

All interviewed clinicians encountered SRIU in at least one patient under their care and, according to their knowledge, one of the patients had subsequently died by suicide. Clinicians estimated the prevalence of SRIU as common and becoming more prevalent in recent years. Some clinicians noted that the prevalence of SRIU is likely to be underestimated:

‘It might be more, way more common than what we're aware of, but [ … ] the first person you'd tell about it wouldn't necessarily be mental health professionals because they can actually do stuff to, you know, intervene.’ (P9, clinical psychologist)

Apart from patients’ potential worry that the disclosure of SRIU might lead to an intervention (such as admission to hospital), clinicians hypothesised that the underestimation of prevalence of SRIU among patients was because of a lack of enquiring about SRIU. This is explored more in Theme 2, *disclosure of SRIU*.

Clinicians encountered various types of SRIU. These included potentially risk-enhancing use, such as researching and acquiring suicide methods online and using social media to facilitate suicide pacts, as well as potential preventative use, such as joining support communities, sharing experiences with others and searching for help online.

#### Researching suicide methods

The most common type of SRIU patients disclosed to the clinicians in our sample was researching suicide and/or self-harm methods. This was most often obtained through internet searches (‘Googling’) and, to a lesser extent, through online communication with other suicidal individuals. In clinicians’ experience, the most important aspects of researching methods for patients were determining pain levels of the method, its efficacy and the quantity of substances to be taken for self-poisoning. Clinicians also reasoned that patients may use the internet to seek replacement for a method used in their previous suicide attempt:

‘Maybe they used methods in the past that hadn't worked for them. Um, and they wanted to find new ways. Maybe more successful ways of taking their own life. Um, and they'd use the internet, so just things like Google searches.’ (P6, clinical psychologist)

As clinicians were aware of the plethora of information on various suicide methods available online, all viewed researching suicide methods as a risk behaviour.

### Theme 2: disclosure of SRIU

This theme explores clinicians’ experiences and beliefs with respect to enquiring about SRIU in their patients.

#### SRIU is not enquired about

All clinicians reported they do not routinely ask their patients about SRIU, and were not aware of their colleagues doing so either. Several clinicians attributed this to the fact that enquiring about SRIU is not a part of standard risk assessment nor was it a part of their training. In addition, some mentioned that they would expect SRIU to emerge naturally in the conversation if it was important to the patient:

‘I would see it as, um, something that would come out naturally in the interview that if a person is using social media as a, as a mean of connection, when I'm talking to about friendships and family relationships [ … ] and if that was linked to their thoughts of self-harm and suicide, then I think I would see that as a natural connection that would've emerged.’ (P4, consultant psychiatrist)

#### Spontaneous disclosure

Clinicians reported that disclosure about SRIU would usually occur when patients talked about methods they had used in a suicide attempt or to self-harm, or those they would use to complete suicide. The most common question to elicit this disclosure was: ‘Why this method?’ The patients volunteered this information willingly when asked about plans to end their life and researching methods online, as illustrated in the following quote:

‘You'll ask about do they have access to those means or do they have an intent to, you know, carry that out. So, they might be less likely to tell you about those things [intent], but usually they're quite open about, um, researching of means.’ (P5, clinical psychologist)

In some cases, patients did not disclose their SRIU directly, but clinicians were aware of it either through case notes or, informally, through another member of the care team or family.

#### Asking about SRIU: a standard/mandatory question?

Most clinicians felt it would be useful to ask their patients about SRIU. They expressed a wish for a standardised question to be part of the risk assessment, listing ubiquity of internet use in general, a need to have this information as an indicator of suicide risk and a variety of other risks and benefits of the internet, as summarised in this quote:

‘Honestly [I] think that it needs to be almost a mandatory question because it's a question that doesn't routinely, I would say, get asked. Um, yeah, one, because of the number of people that no doubt use the internet when they are feeling this way. Um, two, because it could help to formulate what's going on for someone, it could be a really helpful coping strategy, so it could be an intervention that can be harnessed and, and we could, um, kind of build on someone's resources, but also if it's particularly unhelpful, it could be part of someone's formulation as how does this contribute to someone's distress in that moment, how does it make them vulnerable to suicidal thoughts?’ (P2, clinical psychologist)

Clinicians who considered that it was not useful to ask about SRIU viewed current questions about suicidality as sufficient to discern risk. Some clinicians expressed hesitancy about asking these questions directly out of fear it would feel intrusive or spark curiosity about SRIU:

‘I suppose we tread the fine line because [ … ] we're trying to elicit information from the person without giving them hints or tips. So, we wouldn't necessarily mention the internet. Yeah, because what we wouldn't want to be doing is giving them ideas, of course. Or if they haven't already done that, then we wouldn't necessarily want to possibly be seen to suggest that that would be a good idea.’ (P10, mental health nurse)

### Theme 3: individual differences influence disclosure

The third theme illustrates how the age of both the clinician and patient can influence enquiry and disclosure of SRIU. Clinicians were more likely to ask younger patients about their SRIU as they perceived them to use the internet more in general and to be more susceptible to negative influences online. Some clinicians recognised the interaction between age and computer literacy to be of importance.

Age of clinician was also perceived as both a facilitator and a hindrance in asking about SRIU. Some practitioners seemed to be less attuned to the experiences of ‘younger people’ and felt their age prevented them from engaging in these conversations and relating to their experiences: ‘It may be that the generation of psychiatrists below me, that is something that will be much more at their fingertips and they're much more familiar with’ [P4, consultant psychiatrist]. Other practitioners felt their age had facilitated the disclosure:

‘I dunno if this is a bit ageist, but I do think my age helped a little bit as well because a lot of the other [staff members] were like in their forties and fifties. I'm in my, at the time was in my early twenties. Um, and so it's easier for me to build a rapport because I kind of have an understanding of the cultural context that some of the, like younger people were coming from, especially so like people who were maybe up to like 35. [ … ] So, it was easier to build a rapport with that kind of group of people who would be on the internet.’ (P8, care support worker)

### Theme 4: awareness of researching means online as an opportunity to intervene

The fourth theme elaborates clinicians’ thoughts and insights on how knowing that a patient is researching means online can inform care and suicide prevention.

Based on their experience, clinicians hypothesised that researching means comes between thoughts and intent in the suicide process. They pointed out that researching methods is an active action that should indicate a warning sign. Further, clinicians were concerned that gaining information on suicide methods could shorten the period between thoughts and attempt and could even instigate suicidal thoughts in vulnerable patients who were struggling but not necessarily suicidal:

‘I do also think that if someone's, um, maybe quite impulsive and they're researching methods, um, and let's say, you know, something has triggered them that day, you know, well, they've already got those, those thoughts in [their] mind of what they've seen online. It might take less for them then to come up with those plans and have that intent.’ (P8, care support worker)

However, it was also mentioned that knowing a patient was researching suicide methods could ‘buy time’ to intervene:

‘So, if you know that somebody's researching methods online, they might not have made a specific plan. They might not have the intent at that point, but the fact that they are doing that shows that the risks are increasing. It would be useful for them to, to be able to incorporate that into like, let's say the safety plan or to bring it to the [team] and discuss it with the team. So, you know: “What can we do for this person? We know that the risks are increasing. What can we do to help that?”’ (P6, clinical psychologist)

Clinicians believed that a timely awareness of researching methods could help inform interventions and potentially save lives.

### Theme 5: duality of SRIU

The final theme explores clinicians’ experiences with SRIU of their patients that illustrate the duality of SRIU.

#### Online communities as ‘safe spaces’ for patients

Many clinicians mentioned benefits to patients that stem from finding a community online, such as destigmatisation of mental illness experiences and suicidality and sharing one's experience, thus potentially reducing suicidality:

‘[The patient] also found it beneficial, I would say, in terms of sort of the normalisation of [their] experience, meeting people with similar experiences, similar traumas to [the patient], richness of connection and community that the [patient] I was working with in this community did have. Um, so I think she would say on balance, it was an overwhelming positive.’ (P4, consultant psychiatrist)

Clinicians perceived these types of communities as especially valuable to patients who lack social support offline or are experiencing self-stigmatisation. In addition, clinicians acknowledged that sharing stories of recovery from suicidality can be invaluable to inspire hope and recovery in patients.

#### Using the internet instead or as a supplement to offline service provision: risks and benefits

Clinicians mentioned instances where the internet was used instead of or as a supplement to mental health services; for example, when patients were not satisfied with service provision, when access to care was delayed or when they were in crisis. They also mentioned lack of available appointments, which often led patients to turn to the internet:

‘More and more people [are] struggling to access services because services bar will increase an increase and increase, um, that the internet will be a resource that people will have to turn to whether they want to or not, in order to get support.’ (P2, clinical psychologist)

Clinicians often recommended helpful online resources that they knew of and trusted to their patients (available in Supplementary Appendix 2). Clinicians also felt the internet was useful for signposting information and support for both mental health issues (e.g. telephone numbers or chat services when experiencing acute distress) and life in general (e.g. finding a job, accessing benefits).

However, clinicians expressed concern about the quality of other easily accessible information and the detrimental impact it could have on a suicidal patient stating: ‘anybody can post anything, uh, and anybody can access anything’ [P9, clinical psychologist]. Perceived control over what the patient was viewing and the established quality of the online information were the most important factors in recommending online resources. Clinicians were also aware of the possibility to reach out for support from individuals online who might pass wrong information on or have malicious intent and the potential of this to increase suicidality in patients.

‘If people are reaching out for support, they're incredibly vulnerable in, in that moment. And how people respond, who perhaps, um, who perhaps might not be the best people to respond might inadvertently make that experience worse.’ (P2, clinical psychologist)

However, clinicians were aware that receiving appropriate and timely help was not guaranteed when accessing mental health services and suggested that high-quality online support resources could help address this.

#### Encountering harmful content inadvertently or when searching for help

Clinicians were aware of the dangers of coming across harmful suicide-related content accidentally. Some were aware of the possibility of social media algorithms showing harmful content even when it was not explicitly being searching for. In addition, it was mentioned that SRIU could result in harm even when the patient had intended to access help:

‘I've had clients who were like: “Yeah, I went on the internet to help myself and it just triggered me and I felt worse”. Um, so I think it's kind of like a double-edged sword and it's hard to kind of find that balance.’ (P8, care support worker)

Clinicians talked about instances whereby inadvertently viewing suicide-related content could lead to a further deterioration among vulnerable patients:

‘I think if some people are already feeling a little bit off, but they weren't necessarily thinking suicide, I believe it could trigger something. [ … ] I believe that seeing that kind of content when you haven't searched for it can be very, very scary and I believe it can actually trigger up slight deterioration in mental state because you weren't prepared for it.’ (P9, clinical psychologist)

Some were also aware of online suicide prevention ‘pop-up’ adverts that display crisis help information together with suicide-related content. Attitudes towards these were mixed, with some viewing them as helpful and others as lacklustre: ‘I think the help for it, you know, to pop up some adverts or whatever, probably is outweighed by the negative stuff’ (P5, clinical psychologist).

## Discussion

### Main findings

Clinicians often encountered SRIU in their patients and viewed this behaviour as becoming more common. However, they did not routinely enquire about SRIU, even though most saw the benefit in doing so and would welcome enquiring about SRIU in risk assessments in the future. The age of both clinicians and patients was seen as a factor that could influence enquiring about SRIU. They reported that, currently, disclosure of SRIU mostly occurred spontaneously during risk assessment and mostly comprised researching methods online. Being aware that patients are researching methods online was viewed both as an indicator of risk and an opportunity for prevention. Clinicians saw SRIU as potentially beneficial to patients in terms of finding communities of individuals with similar experiences and accessing helpful resources, but were concerned that patients may encounter poor-quality resources and harmful content online, which may affect their suicidality.

Researching suicide methods online was the most common type of SRIU disclosed to the clinicians, supporting findings from a quantitative study carried out by the current authors on SRIU in mental health patients who died by suicide;^9^ however, we do not know that this was the most common or impactful behaviour patients have engaged in. A systematic review by Daine et al^8^ found an association between internet exposure and using more violent methods of self-harm; however, this review only focused on studies including young people, who might be more susceptible to this kind of exposure. Furthermore, many case studies have been published detailing unusual methods of suicide combined with evidence of SRIU.^21,22^ This might be because of the increased cognitive availability following researching methods online that makes the patient aware and accepting of more unusual methods, thus making them more likely to use these methods.^23^ However, it is important to note that this perceived prevalence was probably because of the nature of this disclosure occurring naturally during risk assessment when patients are asked about suicide planning and methods, as reported by the clinicians.

Research on the digital footprint left by online searches shows that searches pertaining to suicide methods moderately correlate with deaths by suicide.^24–27^ In this type of research, lags between online searches and suicide deaths can range between 1 and 3 months;^28^ namely, an increase in suicide-related searches can happen up to 3 months before the increase in suicides on a population level. This corresponds to the theme from the current study: *awareness of researching means as an opportunity to intervene.* Because of the potential presence of the aforementioned lag, timely disclosure on researching methods to the clinician has the potential to buy time to intervene. The only other study we are aware of that mentions clinicians’ attitudes on researching suicide methods online recommends looking up methods online as a topic to be explored with patients, especially those who had a history of suicide attempt and/or self-harm.^15^ For clinicians, this highlights the critical importance of proactively addressing and monitoring online behaviours related to suicide as a preventative measure.

Similar to previous studies on SRIU in children and young people, clinicians perceived asking about SRIU as a useful component of assessments.^12,14,15^ Furthermore, our study has identified worries and reservations around enquiring about SRIU that were common in previous research, such as ‘giving patients ideas’, lack of training on enquiring about SRIU and age differences leading to ‘cultural’ differences between patients and clinicians.^12,14,15^ These are all important areas to address to help make clinicians more comfortable initiating conversations on SRIU and online harms in general. This should be addressed at the service level, providing effective training in SRIU enquiry for clinicians. In addition, it is important to note that barriers to SRIU enquiry mentioned by clinicians in this study mirror those from a similar study by Padmanathan et al,^12^ implying little improvement over time.

We found that clinicians were aware of the potential of SRIU to be both a risk and a protective factor; this implies a more balanced view compared to clinicians in the study by Padmanathan et al,^12^ who felt patients were more harmed than helped by engaging in SRIU. Finding an online community of individuals with similar experiences was regarded as mostly beneficial by clinicians. Similarly, studies on mental health discussion forums found that most participants expressed help-seeking motivation to participate in suicide-related discussion with other users who they felt understood them.^29,30^ Even though we acknowledge that clinicians were unable to ascertain the actual ‘safety’ of these spaces, perceived lack of judgement and destigmatisation of experiences of suicidality and mental illness in some online communities can potentially provide an important sense of community.

Attitudes and experiences towards using the internet instead of or as a supplement to mental health services were mixed, with the most common clinicians’ reservations stemming from awareness of harmful and low-quality content existing online. Studies have found online suicide-related help-seeking to be appealing to people who are suicidal, because of its immediate, informal and anonymous nature.^31–34^ Evidence suggests that neutral and suicide prevention webpages dominate search results when suicide-related terms are searched.^35^ However, the quality of prevention resources is still debatable^36^ and seemingly neutral content can provide information on suicide methods that can be copied by suicidal users.^37^ Notably, it seems that the division of behaviours of visiting suicide-related forums into pro-suicide and suicide prevention categories does not seem to significantly predict users’ suicidal behaviour nor ideation.^38^ Finally, formal online mental health help has been perceived by suicidal users as too impersonal, focused on information and lacking novel advice, sometimes causing users to experience hopelessness.^34^ This implies that the clinicians’ recommendation of formal charity- and healthcare-provided online resources might not satisfy the needs of all suicidal patients.

Some clinicians were also aware of a ‘suicide prevention result’ (SPR) launched in 2010 by Google, an algorithm that displays the telephone number for a suicide prevention hotline as the top result for suicide-related searches.^39^ Even though a welcomed step from the most popular search engine, SPR is not without its shortcomings, as it does not take into account the previous search history of the user nor influence future suicide-related searches.^40,41^ It also does not recognise common risk factors for suicide, for example, the SPR is not displayed when a user searches for media stories on celebrity suicides.^36,42^ In addition, a study that emulated suicide-related Google searches in the USA pointed out lower levels of SPR display when a drug-related term was added to the search (‘how to commit suicide’ versus ‘how to commit suicide [drug name]’).^43^ This is problematic, since these types of search queries may indicate acute crisis and the availability of a potentially highly lethal drug to the user.

### Strengths and limitations

To our knowledge, our study was among the first to specifically investigate clinicians’ experiences and attitudes on SRIU disclosure. The results provide important information on the lack of direct enquiry on SRIU as well as confirmation of potential barriers for it, which could help inform training for clinicians on SRIU. The interview topic guide was developed to capture SRIU in its broadest definition, as any ‘use of internet for reasons relating to an individual's own feelings of suicide’.^1^ This has resulted in both strengths and limitations of the study. As there is a paucity of research on clinicians’ views on SRIU, we felt it was important to allow clinicians to describe all types of SRIU they have encountered in their patients without being limited by more narrow definitions of SRIU or focusing only on risks or benefits. This has resulted in some types of SRIU being more thoroughly discussed than others, such as researching means online, as this was the type of SRIU most commonly encountered by the clinicians. Hence, our data does not explore other types of SRIU in depth. Existent clinician knowledge of SRIU is likely influenced by questions currently included in risk assessment, that is, around planning and methods. Our previous research on SRIU in mental health patients who died by suicide also showed researching means to be the most prevalent type of SRIU; however, it is important to note that the information on SRIU in mental health patients for both pieces of research was provided by clinicians.^9^ Therefore, it remains unclear if these findings represent an actual higher prevalence of researching suicide means online or the relative ease of this type of SRIU disclosure during risk assessment as it is linked to questions on suicide planning and methods routinely asked in risk assessment.

It is possible that clinicians who volunteered to participate have more interest in SRIU for reasons that were not explored in the scope of this study. However, this limitation was partially mitigated by inviting all clinicians to participate, regardless of whether they had experienced SRIU among their patients.

### Implications and further research

Researching means online was the most common type of SRIU that patients spontaneously disclosed to clinicians. Since it takes time to acquire means, especially for more uncommon methods, timely disclosure to one's care team can be a potentially crucial opportunity for intervention. A brief enquiry into the reason for choosing the particular method seems to have the potential to elicit disclosure of SRIU, and recording this within the patient clinical notes could aid prevention. As clinicians in this study mostly reported on researching means online and the disclosure surrounding it, more research is needed to support enquiring about other types of SRIU as it can reveal patients’ potential support systems, sources of information about their condition and/or help-seeking preferences.

Clinicians indicated younger age of patients as indicative for enquiring about SRIU. It is important to note that by using this approach a practitioner is likely to miss both potential risks and potential benefits of SRIU in a significant portion of patients. Training for clinicians on SRIU should therefore emphasise that, even though more prevalent in younger people, SRIU can be present in all age groups. An element of training could also focus on the clinicians’ own age as a potential factor in disclosure, as our results suggest that younger clinicians felt more at ease in these kinds of conversations than older clinicians.

Verified and high-quality online resources on suicidality and mental illness should be available to clinicians to recommend and signpost to their patients. These resources must be designed with suicidal users’ needs for responsiveness and immediacy in mind^34^ as well as updated regularly with new evidence. The currently refined and developed ‘#chatsafe’ guidelines aim to facilitate safer peer communication on suicide online.^44–46^ These guidelines have the potential to be used and recommended by clinicians to patients who have expressed that they are engaging in SRIU.

The online world can enrich the patient's experience, providing community support and resources beyond traditional boundaries of care, as well as invite significant risks. Therefore, a direct, non-judgemental enquiry about whether a patient is engaging in SRIU is a crucial first step in mitigating harmful and promoting beneficial SRIU. Comprehensive training of clinicians on how to balance between enquiry and reluctance to point patients towards potentially harmful content is needed. Giving clinicians the tools to navigate these conversations is important for effective patient care in the 21st century. As clinicians learn about and integrate enquiry on SRIU in standard practice, they can help their patients, regardless of age, navigate online spaces in a safer way, ensuring comprehensive patient support.

## Supporting information

Bojanić et al. supplementary material Bojanić et al. supplementary material

## Data Availability

The data that support the findings of this study are available on request from the corresponding author, L.B. The data are not publicly available because they contain information that could compromise the privacy of research participants.
