# Supplementary material for: Suicide-related internet use of mental health patients: what clinicians know
Source: BJPsych Open. 2024 Nov 5;10(6):e195. doi: 10.1192/bjo.2024.793 (PMC11698162; doi:10.1192/bjo.2024.793)
Supplement: Bojanić et al. supplementary material [file S2056472424007932sup001.docx]

**Appendix 1.**

Table 1. Interview schedule questions/topic guide

| Questions |
| --- |
| 1. Have you ever encountered suicide-related internet use in a patient you were or are currently treating? |
| If yes:   - How did you learn of this patient’s suicide-related internet use? - What was the outcome? |
| 2. What is your usual practice in asking about suicide-related internet use? |
| 3. Do you think asking about suicide-related internet use during an assessment could be useful? In what way? |
| 4. How do you see the link between suicide and suicide-related internet use? |
| 5. What are your views on the risks SRIU for suicidal patients? What do you consider are the benefits of SRIU for suicidal patients? |
| 6. Did you or would you ever recommend to one of your patients to go online while feeling suicidal? What would you or would not recommend? |
| 7. Are there any online suicide prevention resources you know about and trust and/or view as useful? |

**Appendix 2. Online resources clinicians trust and recommend**

| Samaritans | <https://www.samaritans.org/> |
| --- | --- |
| Staying safe | <https://stayingsafe.net/> |
| Get self-help | <https://www.getselfhelp.co.uk/> |
| Mind UK | <https://www.mind.org.uk/> |
| NHS | <https://www.england.nhs.uk/mental-health/> |
| Rethink | <https://www.rethink.org/> |
| Silver Cloud | <https://www.silvercloudhealth.com/uk> |
| CCI | <https://www.cci.health.wa.gov.au/Resources/Looking-After-Yourself> |
| Talk to Frank | <https://www.talktofrank.com/> |
| CalmHarm | <https://calmharm.co.uk/> |
| Bipolar UK | <https://www.bipolaruk.org/> |
| Hearing Voices UK | <https://www.hearing-voices.org/> |
